# Toxicological Mechanism of the Size–Form Synergy of Nano-Copper Oxide in *Danio rerio*

**DOI:** 10.3390/biology14101408

**Published:** 2025-10-14

**Authors:** Qi Liu, Xiaoxuan Li, Lixin Fang, Yanhui Wang, Fang Sun, Peng Liu

**Affiliations:** College of Life and Science and Technology, Harbin Normal University, Harbin 150025, China; liuqi@hrbnu.edu.cn (Q.L.); xiaoxuan.li@nuhighbio.com (X.L.); 16645082899@163.com (L.F.); 13613668464@163.com (Y.W.)

**Keywords:** copper oxide nanoparticles, *Danio rerio*, acute toxicity, anti-oxidation system, micronucleus

## Abstract

**Simple Summary:**

The widespread industrial use of copper oxide nanoparticles (CuO-NPs) has inevitably led to their release into aquatic ecosystems. However, the toxicological effects of CuO-NPs, that is, its biological toxicity, particularly its correlation with particle size and morphology, remains inadequately studied. This study employed zebrafish to evaluate the toxicity of CuO-NPs with varying particle sizes and morphologies. Organ-specific damage was observed in gills, livers, and intestines. Flake-shaped CuO-NPs (SC) exhibited greater toxicity than spherical particles, with smaller particle sizes showing increased toxicity. The primary mechanisms involved reduced tissue antioxidant capacity and diminished microbial α-diversity. Changes in the relative abundance of dominant taxonomic groups at phylum and genus levels contributed to multi-organ toxicity. These findings lay a foundation for the study of the toxicity mechanism of nanomaterials.

**Abstract:**

CuO-NPs demonstrate significant potential across biomedical, environmental protection, and electronic technology domains. This widespread utilization inevitably leads to their discharge into aquatic ecosystems. Research on the biotoxicity of CuO-NPs constitutes a current scientific priority; however, toxicological impacts related to particle size and morphology remain inadequately documented. The zebrafish (*Danio rerio* Roloff, 1956) is employed as a model animal organism to assess acute and subchronic toxicity of differentially sized/shaped CuO-NPs. Organ-specific damage manifested in the gills, liver, and muscles. It was found that sheet-shaped CuO-NPs (SC) could induce the most severe histomorphological alterations. Among spherical CuO-NPs (SP), smaller particles exhibited higher toxicity (SC > 40 nm SP-S > 150–250 nm SP-L). Tissue antioxidant capacity followed the same decreasing trend. The three CuO-NPs in the present study reduced microbial alpha-diversity. Altered relative abundance of dominant taxa is observed at the phylum and genus levels. These results expand toxicological datasets for nanomaterial–vertebrate interactions and support environmental risk assessment for nano-pollutants in natural conditions.

## 1. Introduction

Nanomaterials refer to materials with a size of at least one dimension in the nanometer scale in a multi-dimensional space environment [1]. Compared with ordinary bulk materials, nanomaterials have special physical and chemical properties, such as a large specific surface area, interface effect, etc. [2]. There are many kinds of nanomaterials, which can be divided into carbon nanomaterials (such as single-walled carbon nanotubes), zero valent metal nanomaterials (such as nano gold, nano silver, nano iron), metal oxide nanomaterials (such as nano copper oxide, nano zinc oxide), semiconductor materials (such as quantum dots), and nano polymers (such as polymer latex particles), according to their chemical compositions. As a kind of metal oxide in nanomaterials, CuO-NPs are characterized by black monoclinic crystals or black to brownish black amorphous crystalline powder, with a melting point of about 1330 °C and a relative density of 6.3~6.49 [3]. In addition, they have special magnetic, optical, thermal, mechanical, and acoustic properties.

CuO-NPs show significant potential in biomedical, environmental, chemical engineering, and electronic technologies, particularly as ideal materials for optics, electronics, and sensors [4,5,6]. Additionally, they serve as wood preservatives; antibacterial coatings; gas mask components; colorants for stained glass, ceramic glazes, and artificial gemstones; as well as catalysts, supports, and solid propellants [7]. They can also eliminate biofilms in water pipelines and food processing interfaces [8]. Agricultural applications under development include use as fertilizers, plant growth stimulants, and nanopesticides [9]. Due to their widespread production and accessibility, CuO-NPs inevitably enter aquatic environments [10], dispersing and accumulating through food chains, impacting both freshwater and marine ecosystems [11]. In aquatic systems, their high reactivity drives interactions with various media, altering physicochemical properties (e.g., surface charge and hydrophobicity) and triggering transformation processes [12], thereby modifying their environmental behavior and ecotoxicity in natural conditions. After ingestion by organisms, some CuO-NPs are absorbed via the digestive system, while others accumulate in the intestines or digestive glands. For example, *Daphnia magna* (Straus, 1820) ingests CuO-NPs within 10 min of exposure, showing dispersed particles in the midgut lumen; nanoparticles become observable between microvilli after 48 h [13]. CuO-NPs also accumulate in the digestive glands of *Mytilus galloprovincialis* and the gills/digestive glands of *Mytilus edulis* (Carl Linnaeus, 1758) [14,15].

CuO-NPs also impair the central nervous system in mice; induce hemolysis in rainbow trout (*Oncorhynchus mykiss* Johann Friedrich Gmelin, 1792) [16,17]; and reduce reproductive success, prolong parturition, and increase mortality in guppies (*Poecilia reticulata* Wilhelm Peters, 1859) [18,19].

The zebrafish is a small tropical fish native to South Asia and introduced globally that shares a high genetic and physiological similarity with humans. Compared to mammalian models (e.g., mice/rats), it offers advantages for observing in vitro fertilization, embryonic development, and toxicology studies due to its small size, rapid growth, short sexual maturation, high fecundity and suitability for assays at all developmental stages [20]. It is an ideal model organism for studying the toxicology of nanomaterials. Nanomaterial impacts on zebrafish include embryotoxicity that delays hatching; spinal curvature; pericardial edema; abnormal swim bladder development; yolk sac deformities and hepatic malformations, potentially leading to death. Neurotoxicity results in morphological brain alterations, changes in neurochemical compositions, dysregulated memory-related gene expression, and behavioral impairments; in addition, digestive toxicity is found upon ingestion, with nanomaterials absorbed in the intestines, disrupting neural regulation, inducing inflammation and gut microbiota dysbiosis [21,22]. Nanomaterials subsequently translocate to the liver, accumulating and leading to hepatocyte pyknosis, karyorrhexis, rupture, necrosis, transcriptional dysfunction, metabolic enzyme suppression, oxidative stress, and apoptosis—ultimately compromising liver structure and functions [23].

The present study utilized zebrafish as a model organism to evaluate the acute toxicity of three CuO-NPs with distinct particle sizes and morphologies, followed by a comparative analysis of their toxicological profiles. Based on the 96 h median lethal concentration (96 h-LC_50_), a dose group was established for a 30-day subchronic toxicity study, assessing the impacts of these CuO-NPs on zebrafish genetic toxicity, growth, development, tissue-specific biological functions, and associated damage mechanisms. These findings yield critical theoretical and practical insights, enriching the vertebrate toxicology database for nanomaterials, elucidating CuO-NP toxicity mechanisms, informing the design of novel nanomaterials, and supporting environmental safety assessments under diverse exposure conditions.

## 2. Materials and Methods

### 2.1. Characterization of CuO-NPs and Preparation of Suspension

The morphology and particle size of three types of CuO-NPs (40 nm CuO-NP, C2025035, Shanghai Aladdin Biochemical Technology Company, Shanghai, China; 150–250 nm CuO-NP, C1918115, Shanghai Aladdin Biochemical Technology Company, Shanghai, China; sheet-shaped CuO-NP, DXN-KD30S, Daxinong nanomaterials company, Changzhou, China)were characterized using a scanning electron microscope (SEM) at the Experimental Center of Harbin Normal University. Initially, the samples were placed in the sputter coater. The power supply was activated, and after vacuum evacuation, gold sputtering was performed for 40 s. Upon clicking “Vent” to release the chamber pressure, the sample chamber was slowly opened. The three CuO-NPs particles were affixed to the sample stage using conductive adhesive tape, and their positions were adjusted. Following vacuum evacuations, the electron beam was emitted, and imaging was conducted after optimizing clarity. The X-ray diffraction (XRD) patterns of the three CuO-NPs samples were analyzed using an X-ray polycrystalline diffractometer (XRD, PANalytical X’Pert PRO MRD) under Cu-Kα radiation (λ = 1.5406 Å) at 40 kV and 40 mA. The results confirmed that the selected reagents met the experimental requirements. Subsequently, the three CuO-NPs samples were separately mixed with distilled water and subjected to ultrasonic dispersion (power: 100 W) for 1 h using an ultrasonic homogenizer to prepare 1000 mg·L^−1^ stock suspensions. These suspensions were freshly prepared prior to each experimental use.

Three types of CuO-NP were immobilized on the specimen stage using conductive adhesive. After positional adjustment of the samples, the chamber was evacuated to create a vacuum, followed by electron beam emission. Imaging parameters were optimized until optimal image clarity was achieved, with photographic verification subsequently performed.

### 2.2. Acute Toxicity Test

The acute toxicity test was conducted using a semi-static method. A total of 180 zebrafish (90 male and 90 female) from the same batch (body weight: 0.34 ± 0.005 g, body length: 26.26 ± 0.203 mm) were randomly divided into three groups: 150–250 nm spherical CuO-NPs (SP-L), 40 nm spherical CuO-NPs (SP-S), and sheet-shaped CuO (SC) treatment groups, with 60 fish per group. The zebrafish were purchased from a local fish market that is qualified to sell animals under standard breeding conditions which can be used for the laboratory. A preliminary test was performed to determine the concentration range of CuO-NPs. Six concentration gradients (0, 200, 400, 600, 800, and 1000 mg·L^−1^) were established based on the equal interval method. Each treatment group was placed in identical experimental tanks (33 cm × 24 cm × 18 cm), with 3 L of exposure solution at the target concentration added to each tank. Ten zebrafish were housed in each tank. Feeding was withheld for 48 h prior to and during the experiment with blood worms. The exposure solution was renewed every 24 h to maintain stable CuO-NPs concentrations. Survival counts were observed and recorded at 24 h, 48 h, 72 h, and 96 h. A linear regression curve was generated with mortality as the *y*-axis and CuO-NPs concentration as the *x*-axis. The regression equation was derived to calculate the median lethal concentration (LC_50_) values at 24 h, 48 h, 72 h, and 96 h [19].

### 2.3. Subchronic Toxicity Test

The subchronic toxicity test was conducted using 1/100 and 1/50 of the 96 h-LC_50_ values derived from the acute toxicity test for three types of CuO-NPs. Seven experimental groups were established: control group (0 mg·L^−1^, labeled CK), SP-L 1/100 group (6.4143 mg·L^−1^, labeled SP-L-L), SP-L 1/50 group (12.8285 mg·L^−1^, labeled SP-L-H), SP-S 1/100 group (5.3056 mg·L^−1^, labeled SP-S-L), SP-S 1/50 group (10.6112 mg·L^−1^, labeled SP-S-H), SC 1/100 group (2.2424 mg·L^−1^, labeled SC-L), and SC 1/50 group (4.4848 mg·L^−1^, labeled SC-H).

A total of 420 zebrafish (initial body weight: 0.35 ± 0.003 g, body length: 27.94 ± 0.117 mm) were randomly assigned to the seven treatment groups, with 60 fish per group and three replicates (ammonia 0.03 ± 0.02 mg/L, dissolved oxygen 7.1 ± 1.0 mg/L, temperature 25.2 ± 1.2 °C, pH 7.3 ± 0.2, nitrite ≤0.01 mg/L). The 30th day subchronic exposure was conducted in experimental tanks containing 5 L of target-concentration exposure solutions. Throughout the experiment, temperature and physicochemical parameters were maintained within dynamic ranges. Feeding occurred every 12 h, and residual feed and fecal matter were removed via siphon 30 min post-feeding. CuO-NPs solutions were renewed every 24 h. At the 7th, 14th, 21st, and 28th days of the subchronic test, body weight, body length, and respiratory frequency (number of breaths, which means the number of times the gill cover opens and closes per 30 s) were recorded; in addition, changes in the color of body surface were also recorded [24], and measured for each group. Manual observation of changes in body color of test animals were made at the beginning of the experiment and before the end of the 30th day. All data were recorded into Excel for subsequent analysis. All fish-related experimental procedures were approved by the Animal Care and Use Committee of Harbin Normal University (Approval No. HNUARIA2025003). The fish were euthanized by immersion in a sodium bicarbonate-buffered MS-222 solution (methylcarbamate tricaine) at 300 mg/L. After gill movement ceased, the organisms were immersed for 10 min to confirm successful euthanasia, before proceeding with subsequent tissue collection, antioxidant enzyme activity, micronucleus rate, and omics analysis, etc., experiments.

### 2.4. Histomorphological Observation

At the 15th day and 30th day of the chronic exposure protocol, two zebrafish per treatment group were humanely euthanized. Tissues including the gills, liver, and caudal peduncle muscle were surgically excised and immediately fixed in Bousin’s for histopathological processing. Tissue sections from all experimental groups were subjected to hematoxylin and eosin (H&E) staining to evaluate histomorphological alterations under light microscopy and take pictures.

### 2.5. Measurement of Antioxidant-Related Enzymes and MDA Content

On the 7th, 14th, 21st, and 28th day of the subchronic experiment, two zebrafish from each treatment group were euthanized. The brain and liver were dissected, immediately frozen in liquid nitrogen, and stored at −80 °C until analysis. After precise weighing, tissues were homogenized in ice-cold normal saline at a weight-to-volume ratio of 1:9 (g/mL) using a glass homogenizer under ice-bath conditions. The resulting 10% homogenate was centrifuged at 2500× *g* for 10 min in a refrigerated centrifuge (4 °C), and the supernatant was collected. The supernatant was diluted with normal saline to prepare a 1% homogenate. Protein concentration was determined using the bicinchoninic acid (BCA) microplate assay, with absorbance measured at 562 nm.

(1) Superoxide dismutase (SOD) activity (A001-3-2, Nanjing Institute of Biological Engineering): Quantified by the WST-1 method. Absorbance was read at 450 nm using a microplate reader, and results were expressed as units per milligram of protein (U/mg prot).
(2) Catalase (CAT) activity (A007-1-1, Nanjing Institute of Biological Engineering): Measured via the ammonium molybdate method. The formation of a pale-yellow complex was monitored at 405 nm, and activity was calculated as U/mg prot.
(3) Malondialdehyde (MDA) content (A003-1-2, Nanjing Institute of Biological Engineering): Determined by the thiobarbituric acid (TBA) method. The red MDA-TBA adduct was quantified spectrophotometrically at 532 nm, with results expressed as nanomoles per milligram of protein (nmol/mg prot).

### 2.6. Micronucleus Frequency Analysis in Zebrafish Erythrocytes

On the 30th day of the subchronic experiment, three zebrafish per treatment group were selected. Cardiac blood was collected and smeared onto slides. Micronucleus formation was evaluated in zebrafish peripheral blood erythrocytes. Smears were Giemsa-stained (20 min, RT), and 6000 cells per group (3 fields × 2000 cells) were analyzed using light microscopy (Leica DM500, Leica Microsystems, Wetzlar, Germany, 100× oil immersion). Data represent the percentage of micronucleated erythrocytes relative to total counted cells. For quantification, three random microscopic fields per treatment group were analyzed. In each field, 2000 erythrocytes were counted to record cells containing micronuclei. The proportion of micronucleated erythrocytes was calculated and expressed as a percentage (%).

### 2.7. Effect of CuO-NPs on Gut Microbes in Zebrafish

Following 30 days of subchronic toxicity exposure, nine zebrafish per group (control and three treatment groups at 1/50 of the 96 h-LC_50_ concentration) were dissected under aseptic conditions. Intestinal tissues were aseptically excised, gently rinsed with a sterile 1× PBS buffer (pH 7.2) until luminal contents ceased to outflow, then flash-frozen in liquid nitrogen for 15 min and stored at −80 °C.

The intestinal samples from each treatment group were randomly divided into three biological replicates for 16S rRNA microbiome bioinformatic analysis. Sequencing services were outsourced to Shanghai Personal Biotechnology Co., Ltd. (Shanghai, China), with subsequent bioinformatic processing performed post-sequencing.

### 2.8. Statistical Data Analysis

Statistical analysis was conducted using SPSS 20.0 software (SPSS Inc., Chicago, IL, USA), while linear regression analysis and graphical representation were performed with GraphPad Prism Version 8.0 (GraphPad, San Diego, CA, USA). Multiple comparisons were evaluated using Fisher’s Least Significant Difference (LSD) post hoc test. Statistical significance was defined as *p* < 0.05 (significant) and *p* < 0.01 (highly significant). All data are expressed as mean ± standard error (mean ± SE).

## 3. Results

### 3.1. Morphological Characterization of Three Types of CuO-NPs

**Figure 1:**
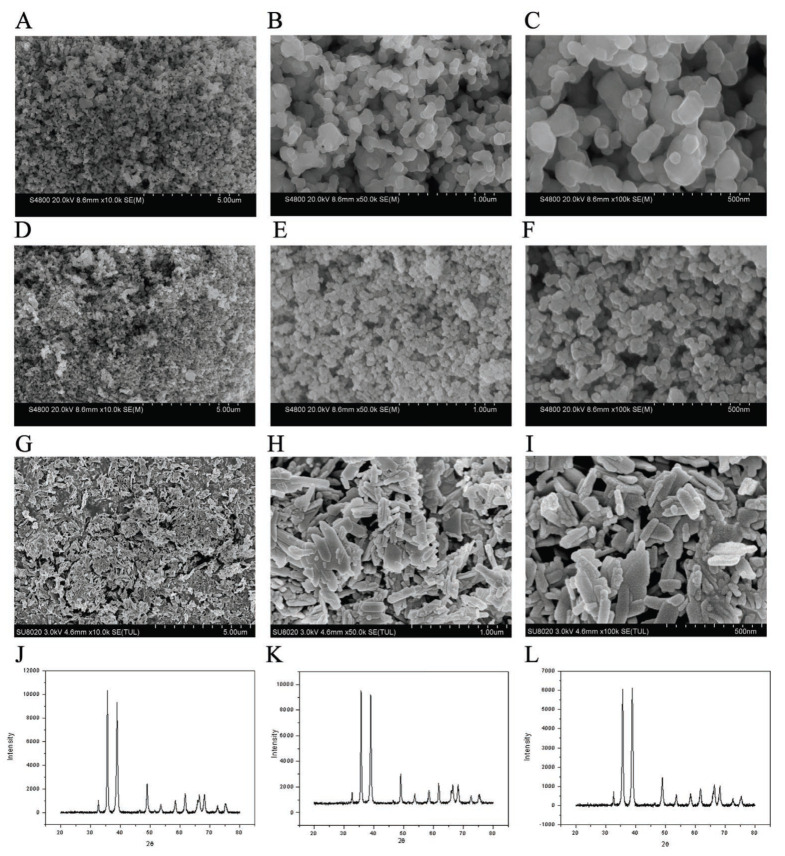
SEM results and XRD patterns of three CuO-NPs ((**A**–**C**) 150–250 nm spherical CuO-NPs; (**D**–**F**) 40 nm spherical CuO-NPs; (**G**–**I**) sheeted CuO-NPs; (**J**) 150–250 nm spherical CuO-NPs; (**K**) 40 nm spherical CuO-NPs; (**L**) sheeted CuO-NPs).

Figure 1A–C shows the morphology of 150–250 nm spherical CuO-NPs after 10,000, 50,000, and 100,000 magnification by electron microscope; Figure 1D–F shows the morphology of 40 nm spherical CuO-NPs at 10,000, 50,000, and 100,000 multiples; and Figure 1G–I shows the morphology of sheeted CuO-NP at 10,000, 50,000, and 100,000. The morphology of copper oxide in each group corresponds with the results of the X-ray diffraction pattern. The results confirm that the selected reagents met the experimental requirements for crystalline phase purity and structural integrity.

### 3.2. Acute Toxicity of CuO-NPs in Zebrafish

**Figure 2:**
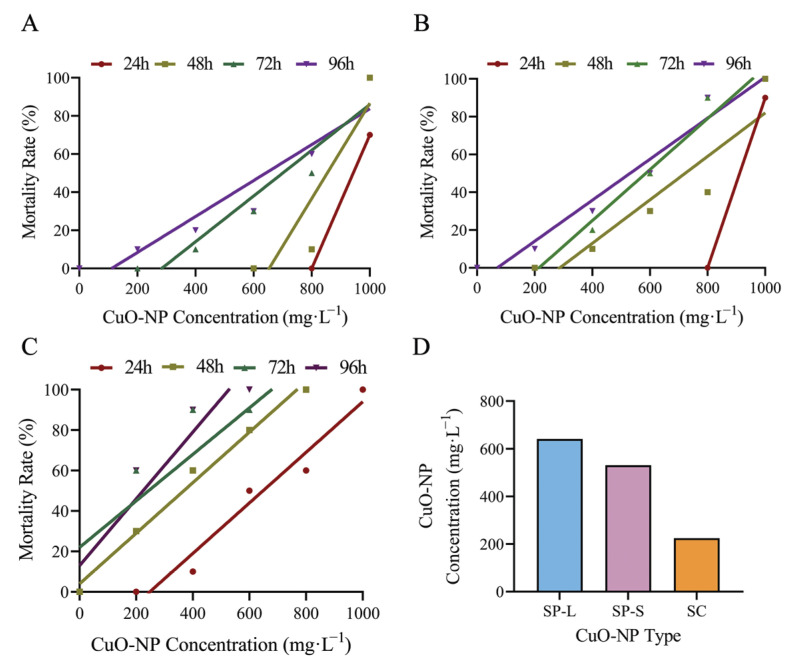
Correlation between concentrations of CuO-NPs and mortality in *Danio rerio* ((**A**) SP-L group; (**B**) SP-S group; (**C**) SC group; (**D**) 96 h-LC50 of three nanomaterials).

Zebrafish mortality exhibited a concentration-dependent positive correlation, with increased mortality at higher concentrations of CuO-NPs (Figure 2). The median lethal concentration (LC_50_) decreased with prolonged exposure duration (Table 1). Notably, the toxicity of CuO-NPs varied significantly depending on particle size and morphology: platelet-shaped CuO-NPs demonstrated the lowest 96 h-LC_50_ (224.24 mg·L^−1^), indicating the highest toxicity, followed by 40 nm spherical CuO-NPs (530.56 mg·L^−1^), while 150–250 nm spherical CuO-NPs showed the highest 96 h-LC_50_ (641.43 mg·L^−1^) and minimal toxicity (Figure 2).

### 3.3. Construction and Evaluation of Zebrafish Model Under CuO-NPs Exposure

**Figure 3:**
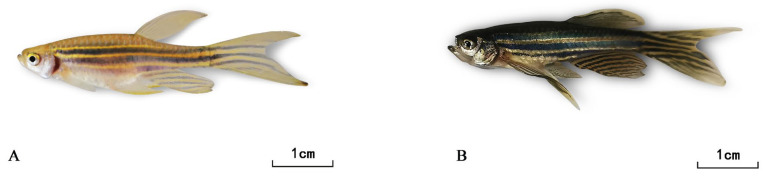
*Danio rerio* with normal body color (**A**) and *Danio rerio* after exposure (**B**).

On the 30th day of the subchronic toxicity study on zebrafish, comparative observations revealed normal behavioral and morphological profiles in the control group, whereas the experimental group exhibited significant toxicological manifestations. These included pronounced hyperpigmentation, which is considered to be a sign of CuO-NP adsorption by the body (Figure 3), elevated stress responses (e.g., erratic swimming patterns with increased velocity), and reduced food intake, consistent with chemical intoxication phenotypes.

### 3.4. Impact of CuO-NPs on Zebrafish Body Length and Weight

The results demonstrated that the average body length in the SC-H group was significantly lower than that of the control group, with a statistically extreme difference (*p* < 0.01). In contrast, other treatment groups showed no significant effects on zebrafish body length (Table 2). This finding indicated that high concentrations of flake-shaped CuO-NPs could markedly inhibit the growth of zebrafish body length.

CuO-NPs exerted a significant impact on zebrafish body weight. A marked decline in weight was observed with prolonged exposure duration: the mean body weights at the 14th, 21th, and 28th day were significantly lower than those at 7th day, with highly significant differences (*p* < 0.01). Furthermore, the SP-S-L, SP-S-H, and SC-H groups exhibited significantly reduced body weights compared to the CK group (*p* < 0.05). Body weight was inversely correlated with CuO-NP concentration, demonstrating that higher nanoparticle concentrations led to lower body weights in the exposed groups (Table 3).

### 3.5. Effect of CuO-NPs on Zebrafish Respiration Rate

Prolonged exposure to CuO-NPs induced dynamic alterations in the respiratory rate of the zebrafish. The highest respiration rate was observed on the 21th day, followed by the 7th, 14th, and 28th days. Under varying treatments, the SP-L-H group exhibited the highest respiratory rate, while the SC-L group showed the lowest. The respiration rate gradually declined with increasing CuO-NP concentrations. Comparative analysis between the control group (CK) and CuO-NP-exposed groups revealed the following descending order of respiration rates: CK group > SP-L group > SP-S group, with the SC group displaying the lowest values (Table 4).

**Figure 4:**
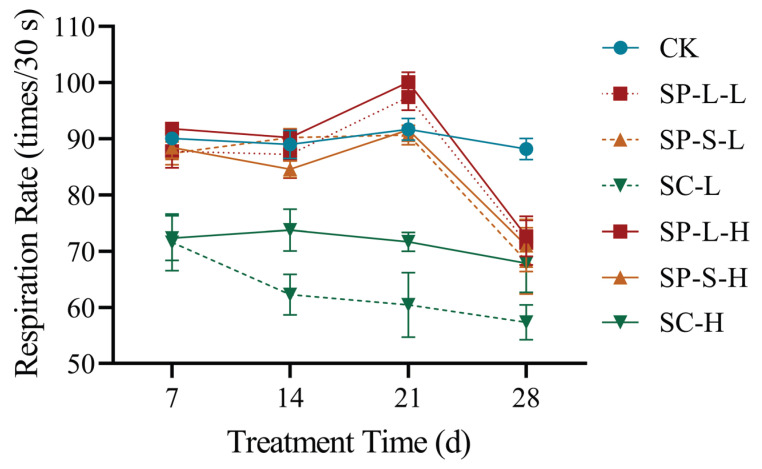
Effects of different CuO-NPs on the respiration rate of *Danio rerio*.

Graphical representation of the respiration rate versus concentration (Figure 4) demonstrated similar trends across groups with identical nanoparticle morphologies but varying concentrations. The control group exhibited a relatively smooth curve from the 7th day to the 28th day. In contrast, the SP-L and SP-S groups displayed an initial slight decline, followed by an increase and, ultimately, a sharp decline. The flake-shaped CuO-NPs group exhibited a consistent downward trend, indicating that flake-shaped CuO-NPs caused the most severe physiological damage to zebrafish.

### 3.6. Histopathological Observations of CuO-NPs on Zebrafish Liver/Gill/Muscle

**Figure 5:**
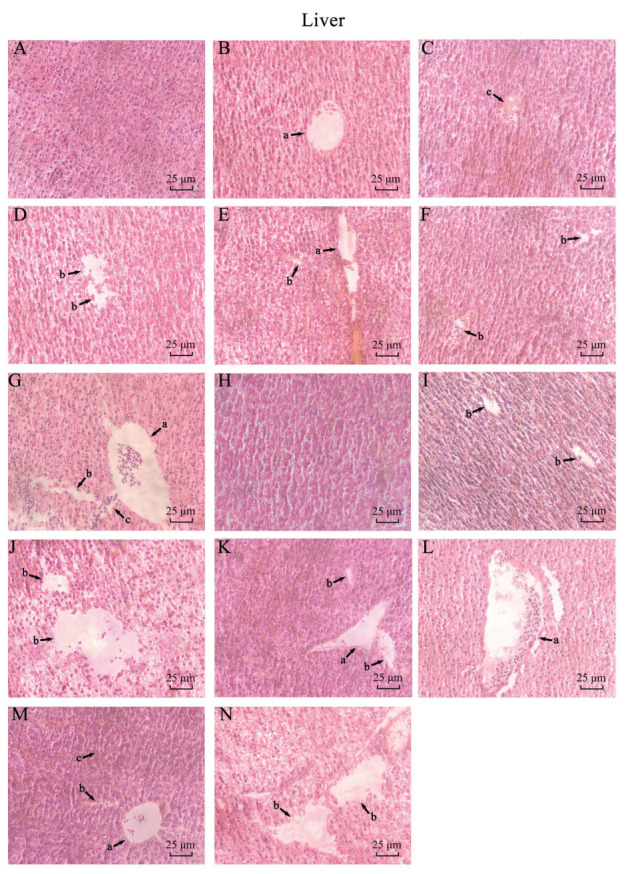
Microstructure of *Danio rerio* liver in each treatment group in subchronic experiment (×400) (Lowercase letters and arrows in the figure: a: hepatobiliary duct; b: inter-tissue cavity; c: connective tissue) ((**A**) 15 d CK; (**B**) 15 d SP-L-L; (**C**) 15 d SP-L-H; (**D**) 15 d SP-S-L; (**E**) 15 d SP-S-H; (**F**) 15 d SC-L; (**G**) 15 d SC-H; (**H**) 30 d CK; (**I**) 30 d SP-L-L; (**J**) 30 d SP-L-H; (**K**) 30 d SP-S-L; (**L**) 30 d SP-S-H; (**M**) 30 d SC-L; (**N**) 30 d SC-H).

The hepatic microstructure of zebrafish after H&E staining is illustrated in Figure 5. In the CK group, the liver tissue exhibited normal architecture with minimal connective tissue and no typical portal triads. Hepatocytes displayed intact morphology, characterized by regularly rounded nuclei, homogeneous cytoplasm, tightly packed cellular arrangements, and narrow intercellular spaces. In contrast, the CuO-NPs treated group demonstrated varying degrees of pathological alterations. These included patchy necrosis within hepatic tissue, irregular hepatocyte alignment, and morphological changes such as nuclear irregularity and partial karyolysis. Additionally, severe vacuolar degeneration, tissue edema, and erythrocyte infiltration were observed in multiple regions.

**Figure 6:**
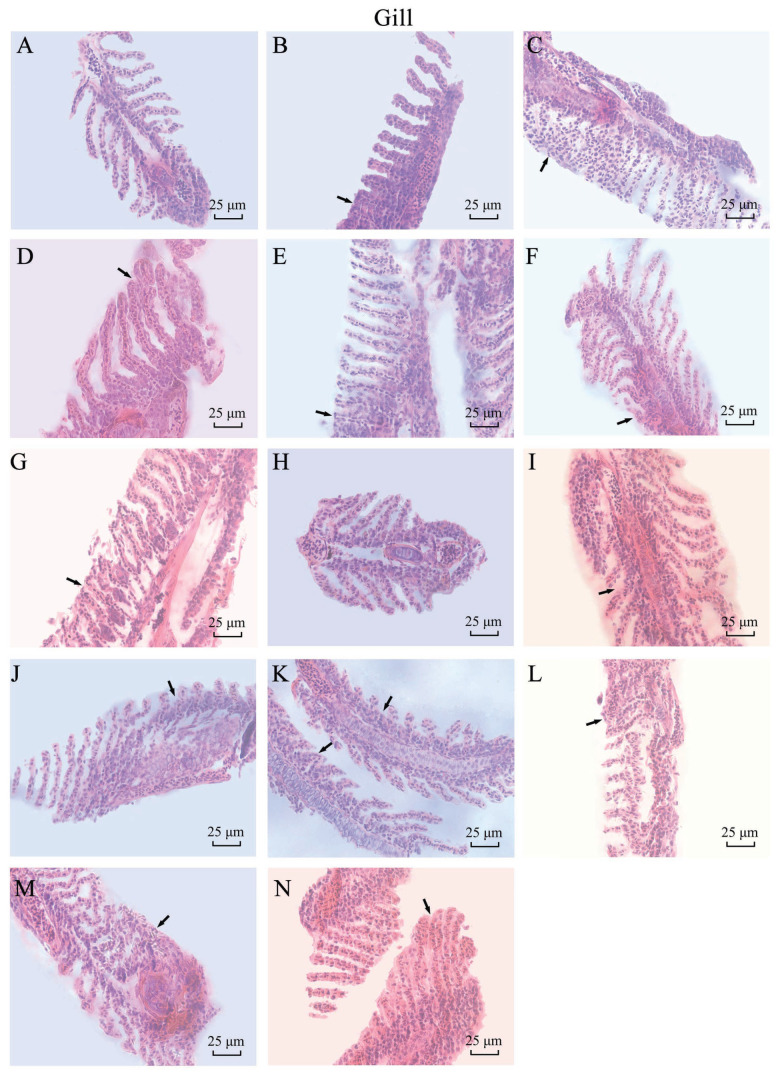
Microstructure of *Danio rerio* gills in each treatment group in subchronic experiment (×400) (The arrow marks the lesion area in the figure) ((**A**) 15 d CK; (**B**) 15 d SP-L-L; (**C**) 15 d SP-L-H; (**D**) 15 d SP-S-L; (**E**) 15 d SP-S-H; (**F**) 15 d SC-L; (**G**) 15 d SC-H; (**H**) 30 d CK; (**I**) 30 d SP-L-L; (**J**) 30 d SP-L-H; (**K**) 30 d SP-S-L; (**L**) 30 d SP-S-H; (**M**) 30 d SC-L; (**N**) 30 d SC-H).

The microstructure results of zebrafish gills after H&E staining are shown in Figure 6. The figure shows that the epithelial cells of the control fish have an orderly arrangement with a round or oval nucleus. Compared with the control group of gill tissue, the CuO-NP treatment group of zebrafish gills appeared to show different degrees of damage and gill small sheet epithelial cell layer swelling; and with the increase of concentration, gill small sheet epithelial cell hyperplasia became gradually obvious and was accompanied by degeneration, shedding, and infiltrating inflammatory cells to fill the whole gill’s small gap and make the gill wire swelling stick, in a lamina fusion phenomenon. According to the results of the zebrafish gill microstructure, the experimental group showed lesions while the control group did not. Additionally, the longer the time and the higher the concentration, the more severe the gill damage is. Among the nano-copper oxide treatment group, the SC group was the most severe, while the SP-L and SP-S groups were mild. This indicates that the sheet nano-copper oxide causes more damage to the gills than the sphere.

**Figure 7:**
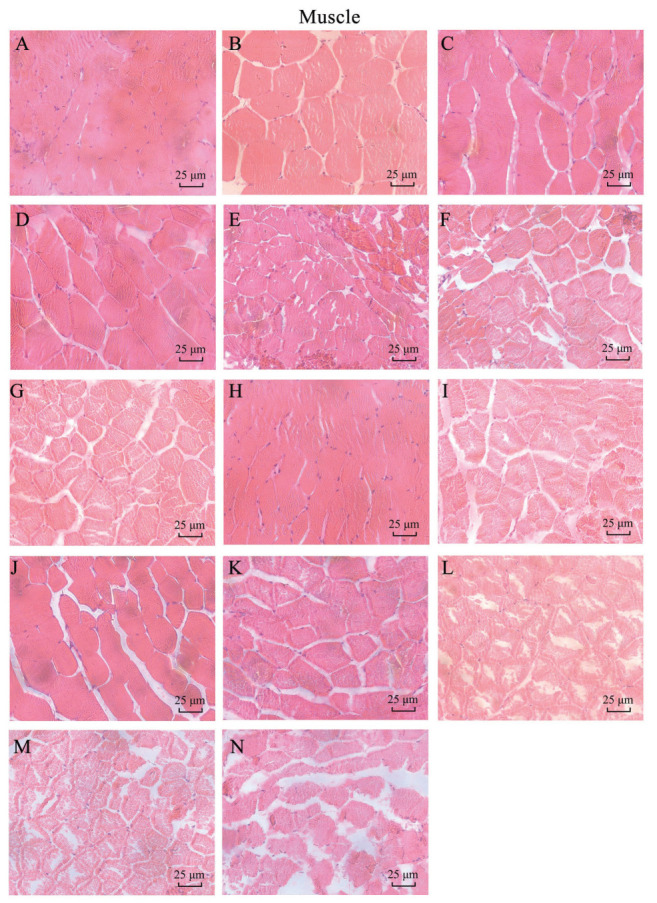
Microstructure of *Danio rerio* muscles in each treatment group in subchronic experiments (×400) ((**A**) 15 d CK; (**B**) 15 d SP-L-L; (**C**) 15 d SP-L-H; (**D**) 15 d SP-S-L; (**E**) 15 d SP-S-H; (**F**) 15 d SC-L; (**G**) 15 d SC-H; (**H**) 30 d CK; (**I**) 30 d SP-L-L; (**J**) 30 d SP-L-H; (**K**) 30 d SP-S-L; (**L**) 30 d SP-S-H; (**M**) 30 d SC-L; (**N**) 30 d SC-H).

The microstructure results of H&E staining of the muscles at the zebrafish tail stalk are shown in Figure 7. According to the figure, the muscle fibers of the control muscles were evenly distributed, with small gaps and a roughly regular round nucleus. In the exposed group, muscle fibers appeared separated, and some muscle fibers broke and disintegrated, with inflammatory reactions appearing. According to the microstructure of zebrafish muscle, the nano-copper oxide treated group showed more damage than the control group. The degree of injury is directly proportional to treatment time and the treatment concentration. In the CuO-NP treatment group, the SC group was the most severe, followed by the SP-L group, and the SP-S group was the lightest. This indicates that for zebrafish muscle damage, the small particle size CuO-NP causes more damage than the large particle size, and the sheet of CuO-NP causes more damage than the sphere.

### 3.7. Impact of CuO-NPs Exposure on Antioxidant Enzymes Activities and MDA Content

#### 3.7.1. SOD Activity

**Figure 8:**
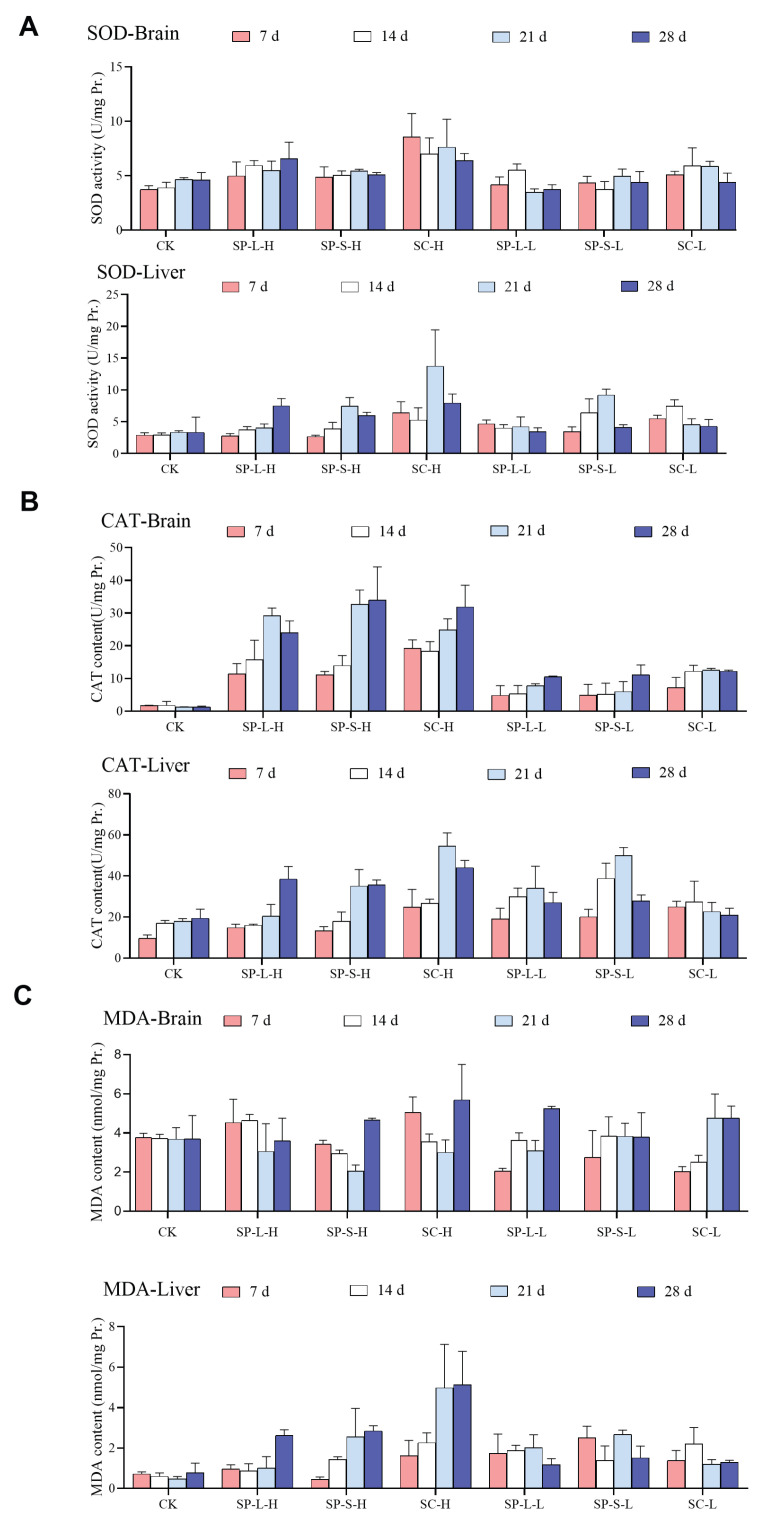
Activity of anti-oxidative enzymes and MDA content in brain and liver. (**A**) SOD activity during CuO-NP exposure; (**B**) CAT content during CuO-NP exposure; (**C**) MDA content during CuO-NP exposure.

For SOD activity in zebrafish brain tissue, the SC-H group was significantly higher than other groups in different groups (*p* < 0.05), with the highest SOD activity showing in the SC group (*p* < 0.05). Comparing different particle sizes, SOD activity of SP-S group was significantly higher than SP-L group (*p* < 0.01), and sheet size was significantly higher than sphere (*p* < 0.01). With the extension of treatment time, the SOD activity of zebrafish liver tissue showed a trend of rising and then decreasing. At the 21st day, the enzyme activity reached the highest value, the SC-H group’s value was significantly higher than that of other groups (*p* < 0.05), and the concentration also caused an increase in enzyme activity, with the SC group under a different nano-copper oxide, which was significantly higher than that of other groups (*p* < 0.01) (Figure 8A).

#### 3.7.2. CAT Activity

The activity of CAT in zebrafish brain tissue increased with time. Among the different treatment groups, the CAT activity of SP-L-H, SP-S-H, and SC-H was the highest (*p* < 0.01), the increase of concentration increased the enzyme activity significantly, the SP-S group was higher than SP-L group, and the enzyme activity of SC group was higher than the spherical group (Figure 8B). The CAT activity of liver tissue increased with the increase in treatment time, with the highest enzyme activity of the SC-H group in different treatment groups (*p* < 0.05), while the enzyme activity of the SP-S group was greater than that of SP-L group, and that of SC group was higher than spherical group under different morphology (Figure 8B).

#### 3.7.3. MDA Content

With longer exposure time, zebrafish brain MDA peaked at 28 d (*p* > 0.05), with no significant difference between treatment groups (Figure 8C). MDA content in liver tissue accumulated with the increase in treatment time. Under different treatments, the SC-H group had the highest MDA content, the CK group had the lowest content, the MDA content increased significantly with increasing concentration (*p* < 0.05), the SP-S group was higher than the SP-L group, and the SC group was significantly higher than spherical (*p* < 0.05) (Figure 8C).

### 3.8. Impact of CuO-NPs on Micronucleus Frequency in Zebrafish

**Figure 9:**
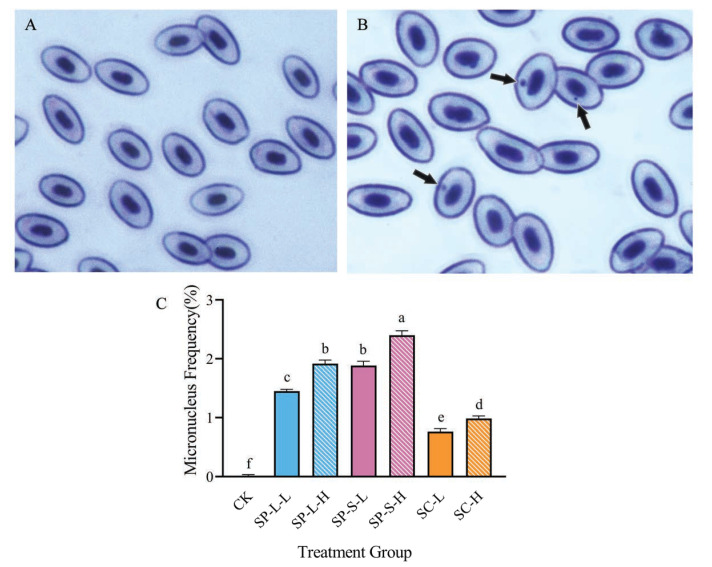
Normal erythrocytes (**A**) and micronucleated erythrocytes (**B**) in *Danio rerio* micronucleus frequency (%) (**C**). Micronucleus frequency rate(%) during CuO-NP treatment. Note: The arrows indicate micronuclei.

Microscopic examination of stained zebrafish blood smears identified both normal erythrocytes and micronucleus-containing erythrocytes (Figure 9A,B). The micronucleus frequency across treatment groups is summarized in Figure 9C. In the control group, micronucleus formation was negligible, whereas all experimental groups exhibited significantly higher frequencies than the CK group. Notably, the SP-S group displayed the highest micronucleus frequency, followed by the SP-L group, while the SC group (specific morphology) showed the lowest frequency. Additionally, high-concentration groups demonstrated elevated micronucleus frequencies relative to their low-concentration counterparts. The SP-S were correlated with higher micronucleus frequencies and greater genotoxicity than the SP-L group. Morphological analysis further revealed that spherical CuO-NPs induced significantly higher micronucleus frequencies and genotoxicity than platelet-shaped CuO-NPs.

### 3.9. Impact of CuO-NPs on Gut Microbiota in Zebrafish

**Figure 10:**
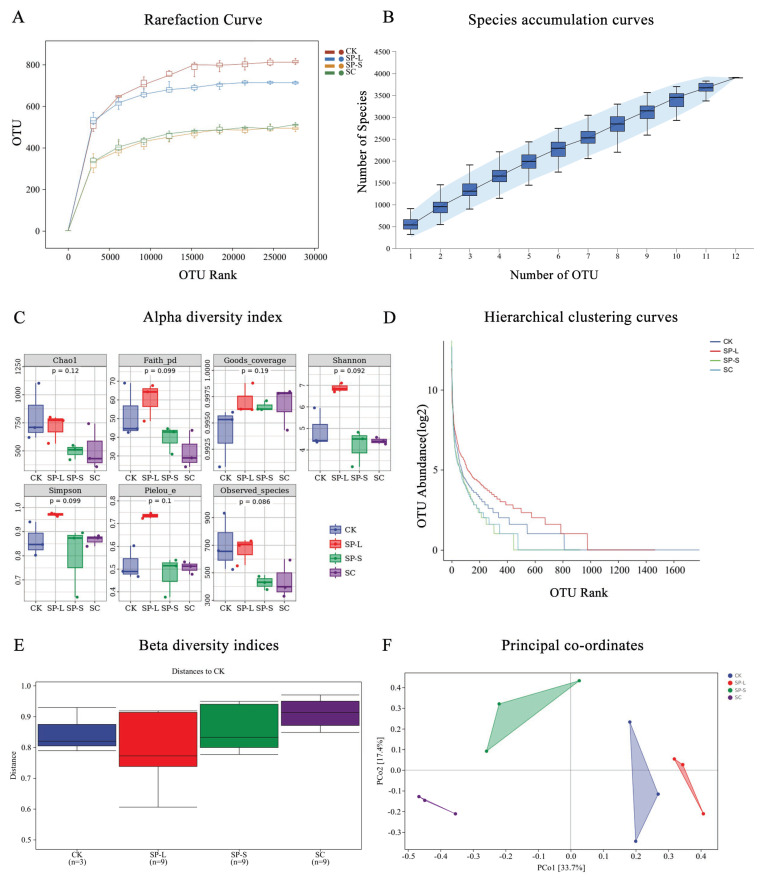
Impact of CuO-NPs on gut microbiota in zebrafish. (**A**) Rarefaction curve of CuO-NP exposure on zebrafish; (**B**) Species accumulation curves of CuO-NP exposure on zebrafish; (**C**) Alpha diversity index of CuO-NP exposure on zebrafish; (**D**) Hierarchical clustering curves of CuO-NP exposure on zebrafish; (**E**) Beta diversity indices during CuO-NP exposure; (**F**) Principal coordinates during CuO-NP exposure on zebrafish.

Rarefaction curves and species accumulation curves of intestinal microbiota across treatment groups are presented in Figure 10A,B. Alpha diversity analysis (Figure 10C) showed no significant intra-group differences across the four treatment groups (*p* > 0.05), suggesting comparable species diversity within each experimental condition. The rarefaction analysis revealed that the estimated total species richness and relative abundance plateaued at the current sequencing depth, suggesting adequate coverage of microbial diversity. The species accumulation curve further demonstrated that community richness increased with sample size but gradually stabilized, indicating sufficient sampling effort. Both curves approached asymptotic trends with the existing sequencing volume, confirming the appropriateness of the dataset for downstream analyses. Hierarchical clustering analysis (Figure 10D) demonstrated distinct community structures among groups. The control group exhibited the broadest horizontal axis span and the most gradual curve slope, reflecting the highest species richness and relatively uniform taxonomic distribution.

In contrast, beta diversity indices (Figure 10E) indicated no statistically significant differences (*p* > 0.05) between treatment groups, implying minimal divergence in microbial community composition among experimental conditions. Principal coordinates analysis (PCoA) of beta diversity (Figure 10F) revealed clear inter-group separation, with the first two principal components explaining 33.7% and 33.3% of total variance, respectively.

### 3.10. Effect of CuO-NPs on Gut Microbe Composition in Zebrafish

**Figure 11:**
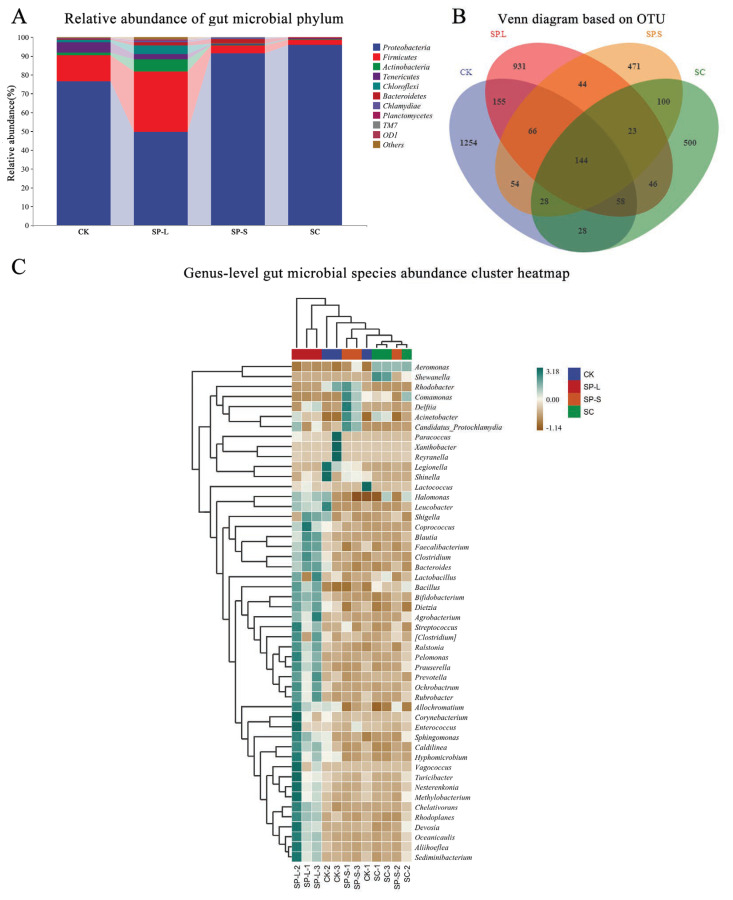
Effect of CuO-NPs on gut microbe composition in zebrafish. (**A**) Relative abundance of gut microbial phylum during CuO-NP exposure; (**B**) Venn diagram based on OTU during CuO-NP exposure; (**C**) Genus-level gut microbial species abundance cluster heatmap during CuO-NP exposure.

The results of top10 clustering analysis at phylum level of zebrafish gut microbial communities after exposure to nano-copper oxide with different particle sizes and morphology are shown in Figure 11. As shown in Figure 11A, according to the results of the relative abundance of top 10, *Proteobacteria* (*proteobacteria*), *Firmicutes* (*firmicutes*), and *Actinobacteria* (*actinobacteria*) are the three major phyla of zebrafish gut, accounting for 78.40%, 13.13%, and 2.27% of the total sequencing volume, respectively. Among them, *Proteobacteria* with the highest abundance in zebrafish gut microbial community decreased in the SP-L group but increased significantly in SP-S group and SC group; *Firmicutes* and *Actinobacteria* increased in the SP-L group and decreased in the other two groups.

In this experiment, OTUs were clustered and analyzed (Figure 11B). The CK group exhibited the highest OTU count, reaching 1787, followed by the SP-L group (1467 OTUs), SP-S group (930 OTUs), and SC group (927 OTUs). Taxonomic uniqueness analysis revealed distinct OTU distributions: the CK group contained 1254 unique OTUs, while the SP-L, SP-S, and SC groups harbored 931, 471, and 500 unique OTUs, respectively. Notably, only 144 OTUs were conserved across all four treatment groups, representing the core microbial flora. These findings indicate that the three Coxides significantly reduced microbial diversity, with the most pronounced reduction observed in the SP-L group.

The results of heatmap analysis of genus-level microbial species abundance of zebrafish gut microbial communities after exposure to CuO-NPs with different particle size and morphology are shown in Figure 11C. The dominant bacteria mainly include *Aeromonas* (*Aeromonas*), *Eryrobacter* (*Rhodobacter*), *Shewanella* (*Shewanella*), and *Oceanicaulis*, etc. Compared with the control group, *Aeromonas* showed an increase in the proportion of each treatment group, and the abundance was significantly different. The abundance of *Oceanicaulis* in the SP-L group was significantly higher than that of the other groups, and *erythrobacter* had the highest abundance in the SP-S group, while *Shewanella* in the SC group was higher than that in the other groups.

## 4. Discussion

In recent years, nanomaterials have demonstrated groundbreaking advancements across multiple disciplines including healthcare, energy and electronic technologies, environmental remediation, and catalytic applications [25]. For instance, multifunctional nanocarrier systems enable targeted delivery of gene therapeutics and antimicrobial agents, facilitating localized drugs release. CuO-NP exhibit selective cytotoxicity toward pathological entities such as neoplastic cells [26]. Furthermore, these materials show promising applications in bioprinting technologies for tissue engineering and vascular scaffold fabrication. Unlike conventional environmental toxicants, standardized toxicological assessment protocols for nanomaterials remain underdeveloped. As nanomaterial utilization expands exponentially, potential adverse effects—particularly concerning long-term biosafety profiles and bioaccumulative toxicity—have become a subject of significant scientific scrutiny.

CuO-NP is a kind of nanomaterial widely used in the fields of environmental and energy catalysis, biomedicine, electronics, and materials engineering [18,27]. The wide application of CuO-NPs leads to its inevitable release to the environment and organisms [19]. Potential biological toxicity has also gradually become a research hotspot. Unlike traditional copper ions, the toxicity of CuO-NPs not only originates from the release of copper ions but is also closely related to its nanoscale effects (such as high surface activity and the ability to penetrate biological barriers). In recent years, researchers have revealed its toxic mechanism through interdisciplinary approaches and tried to reduce risks through material modification. Generally, the biological safety of pollutants in water environments is evaluated, and acute toxicity experiments and chronic toxicity experiments should be considered [28]. However, at present, most classification standards of acute toxicity of toxic substances do not apply to nanomaterials; and due to their size, morphology, surface charge, and a variety of characteristics, there is no way to accurately evaluate the toxicity of nanomaterials. In recent years, researchers have revealed the mechanism of nanomaterial toxicity through interdisciplinary approaches and tried to reduce toxicity by changing the properties of materials (such as particle size, shape, etc.). Previous studies have indicated that subacute inhalation exposure to CuO-NP showed immunomodulatory effects in asthma [29]; CuO-NP has cytotoxic effects on esophageal cancer cells, and also showed antimicrobial and heavy metal sensing activities. Subchronic toxicity experiments reveal the effects of CuO-NPs on zebrafish at non-lethal concentrations [30]. In the present study, it was observed that the zebrafish exhibited darker body coloration, possibly due to poisoning and the adsorption of CuO-NP on the skin. Analysis of zebrafish length, weight, and respiration rate showed that among different particle sizes and shapes of CuO-NPs, smaller particles showed more distinct damage, which indicates that CuO-NPs with smaller particle size has stronger toxicity in growth and development; additionally, plate-shaped particles have stronger toxicity than spherical ones. The changes in respiration rate over time are speculated to be related to the shape of the CuO-NPs, which can adhere to the zebrafish’s respiratory organs, causing breathing difficulties and a decrease in respiration rate. The initial increase in respiratory rate for spherical particles may be due to compensatory breathing, leading to a temporary rise in frequency, followed by a decline due to organ failure. In contrast, plate-shaped particles have stronger adhesion and higher toxicity, resulting in a continuous decline in respiration rate.

The toxicity of CuO-NPs is often associated with the particle size and morphology on its toxicity. The 96 h-LC_50_ of CuO-NPs on some fish has been reported, and the 96 h-LC_50_ of lake carp (*Rutilus rutilus* Linnaeus, 1758), guppies, and common carp (*Cyprinus carpio* Linnaeus, 1758) is 2.19, 28.354, and 124.9 mg·L^−1^, respectively. In the present study, the 96 h-LC_50_ of three CuO-NP in zebrafish was 641.43, 530.56, and 224.24 mg·L^−1^, which confirmed that the zebrafish has a high tolerance to CuO-NP and is a suitable model animal for toxicological experiments. Prior studies have demonstrated significantly elevated copper levels in gills following CuO-NP exposure, which markedly increased SOD and GSH-Px activities as well as lipid peroxidation [31]; the alterations in serum biochemical parameters of fish exposed to sublethal concentrations of CuO-NPs encompass elevated triglyceride levels, concomitant with histopathological lesions in gill and hepatic tissues as well as hydropic degeneration of the liver, demonstrating dose-dependent increases in toxicity [32]; after seven days of exposure to CuO-NPs, bivalves exhibited significantly reduced gill filtration rates, respiration rates, and hemocyte phagocytic responses [33]. In the present study, gills showed wire swelling stick and lamina fusion, with vacuoles appearing in the cytoplasm of the epithelial cells in the gill lamella (H&E shows a translucent area).

The liver is an important organ for toxic metabolism; its histomorphology and biochemical indicators are closely related to its function. Previous studies determined liver functions related to indicators, which indicate that the phenomenon of fish liver functional blood serum indicators (ALT, ALP, LDH, etc.) exposed to 0.05 mg/L CuO-NP for 21 days showed significant abnormalities, accompanied by a decrease in antioxidant enzyme activities and content [34]; Mahmoud et al. indicated that CuO-NP could induce cellular toxicity in liver and intestine cell lines [35]. In the present study, it was observed that exposure to CuO-NPs can induce morphological alterations in the liver, characterized by a loose texture, swelling in some hepatocytes, condensation of cell nuclei, and infiltration of inflammatory cells in specific areas. Furthermore, a notable increase in the levels of MDA (a marker of lipid peroxidation) was detected in the liver, indicating that the liver damage caused by CuO-NPs may be attributed to oxidative stress and the overactivation of lipid peroxidation. The specific molecular mechanism still needs further experimental verification.

Previous studies have shown that CuO-NP has intestine toxicity. Fish intestines harbor a diverse and abundant microbial community, and the relative stability of the gut microbiota is crucial for the host’s health [36,37]. Disruption of the gut microbiota balance can lead to the occurrence of diseases in organisms. Other researchers have also investigated the impact of nanomaterials on gut microbiota [38]. CuO-NP induce non-alcoholic fatty liver disease by disrupting bile acid homeostasis and perturbing the intestinal microbial homeostasis [39]; CuO-NP can be used to help in the treatment of experimental colitis in Swiss albino mice [40]. Research indicates that the effects of nanomaterials on the intestinal microbiota of organisms are mostly harmful, but the pathways through which these effects occur are still unclear. They may also pose threats to human health, necessitating further evaluation of the biological safety of nanomaterials. Adult zebrafish exposed to CuO-NP showed changes in the composition of their gut microbiota at the genus level. In the present study, it was observed that, compared to the control group, CuO-NPs significantly reduced the diversity of intestinal microorganisms and CuO-NPs could notably inhibit the *Pseudomonas* genus, which is beneficial to zebrafish growth to some extent. However, the decrease in *Actinobacteria* abundance might weaken the intestinal barrier function, leading to the occurrence of inflammatory bowel disease. *Firmicutes*, *Proteobacteria* and *Fusobacterium* were dominant microbial communities in adult zebrafish intestines. In the SP-L group, *Proteobacteria* decreased while *Firmicutes* and *Actinobacteria* increased. Conversely, in SP-S and SC groups, *Proteobacteria* rose while *Firmicutes* and *Actinobacteria* declined. The low *Firmicutes* abundance and high *Proteobacteria* levels characteristic of inflammatory bowel disease (IBD) in zebrafish models indicate IBD development in the SP-S and SC groups. The reduced *Actinobacteria* abundance may compromise intestinal barrier function. When adult zebrafish were exposed to nano-copper oxide, their gut microbiota composition underwent significant changes at the genus level. *Aeromonas*, a type of gram-negative short bacillus in the *Vibrio* family, is a major pathogen in fish and can occasionally cause infections in humans and animals, leading to intestinal diseases or food poisoning. Among the four treatment groups, the CK group showed the lowest Aeromonas abundance, followed by the SP-L group, then the SP-S group, with the SC group having the highest levels. This indicates that zebrafish also face increased risks of intestinal diseases. *Pseudomonas*, an aerobic Gram-negative bacterium, forms rod-shaped or slightly curved colonies. Certain strains in this genus can metabolize to produce various water-soluble pigments such as colistin, fluorescein, erythromycin, and black pusin. As a primary pathogen causing hospital-acquired infections, *Pseudomonas* species can lead to multiple diseases in both animals and humans. Results showed that compared with the CK group (27.89%), the three nano-copper oxide treatment groups demonstrated significantly reduced *Pseudomonas* abundance: SP-L (10.46%), SP-S (16.31%), and SC (1.34%). This indicates that nano-copper oxide effectively inhibits *Pseudomonas* growth, which may benefit zebrafish development to some extent. In conclusion, in terms of intestinal microorganisms, nano-copper oxide has a dual effect on the growth of zebrafish, which can easily lead to intestinal inflammation and inhibit the growth of pathogenic bacteria. However, there is no significant difference in diversity index between different nano-copper oxides. Notably, CuO-NPs displayed dual effects on gastrointestinal pathophysiology: while potentiating pro-inflammatory responses, they concurrently suppressed pathogenic bacterial proliferation.

It can be seen that the different particle size and morphology of CuO-NPs have a significant impact on the results of acute toxicity experiments; the sheet CuO-NPs are the most toxic, while 40 nm spherical CuO-NPs are the second-most, and 150–250 nm CuO-NP was the least toxic. It can be concluded that for CuO-NPs, the sheet size is more toxic than the spherical size, and the small size is more toxic than the large size. This may be due to the increased surface area of nanoparticles leading to more active sites and higher ion release rates. Long-term toxic effects are also related to particle size, with smaller nanoscale copper oxide particles showing a more significant inhibitory effect on biological development [41]. Nanoparticles of different morphologies show varying solubility and stability in biological environments. The flaky structure, with a larger surface area, releases Cu2+ more easily, leading to intracellular metal ion overload and enhanced oxidative stress. The histomorphological observations of liver, gills, or muscle showed different degrees of damage during exposure to CuO-NP; injury was positively correlated with CuO-NP concentration. Antioxidant enzyme activity was positively correlated with the exposure concentration. Smaller spherical nanoparticles demonstrated dose-responsive correlations with increased developmental toxicity, manifested through growth retardation, histomorphological alterations, and compromised antioxidant capacity. Comparative analysis showed that laminar particles exhibited significantly higher toxicity than their spherical counterparts across all evaluated parameters. Beyond the observed phenotypic and biochemical impairments, exposure induced dysbiosis in the intestinal microbiota, characterized by disrupted homeostasis and altered microbial abundance.

The present investigation provides substantive contributions to nanotoxicology by elucidating the size and morphology-dependent toxicodynamics of engineered nanomaterials. The findings enrich vertebrate toxicological databases, clarify CuO-NPs toxicity mechanisms, and establish a theoretical framework for rational nanomaterial design. Furthermore, the derived data offer critical insights for environmental risk assessment of nanomaterials under varying physicochemical conditions.

## 5. Conclusions

The toxic effects of CuO-NPs are closely associated with particle size, concentration, exposure pathways, and environmental conditions. Studies indicate that CuO-NPs reduce antioxidant enzyme activity, leading to reactive oxygen species (ROS) accumulation that triggers oxidative stress. This process damages intestinal tissue integrity, disrupting both gut biological functions and the body’s immune system. Furthermore, the bioaccumulation of CuO-NPs may pose long-term ecological risks to aquatic organisms and soil microbial communities.

Although the toxic mechanism has been revealed in this study, the following matters need to be further explored: the chronic toxicity and transgenerational effects of long-term exposure to low concentrations are not clear; the transformation and toxicity of CuO-NPs in complex media (such as organic matter, pH change) need to be further verified; exposure limit standards based on ecology and human health need to be developed. Future research should combine multi-omics techniques (such as transcriptome and proteome) and in situ characterization methods to provide a scientific basis for the safe application of nanomaterials.

## Figures and Tables

**Table 1:** Regression equation and median lethal concentration between three kinds of CuO-NPs concentration and *Danio rerio* mortality rate.

Group	Time (h)	Y = aX + b	Correlation (R^2^)	LC_50_ (mg·L^−1^)
SP-L	24	Y = 0.3500 × X − 280.0	1.0000	942.8571
	48	Y = 0.2500 × X − 163.3	0.8242	853.2000
	72	Y = 0.1200 × X − 34.00	0.9172	700.0000
	96	Y = 0.0943 × X − 10.48	0.8975	641.4254
SP-S	24	Y = 0.4500 × X − 360.0	1.0000	911.1111
	48	Y = 0.1150 × X − 33.00	0.8644	721.7391
	72	Y = 0.1350 × X − 29.00	0.9746	585.1852
	96	Y = 0.1086 × X − 7.619	0.9670	530.5617
SC	24	Y = 0.1250 × X − 31.00	0.9586	648.0000
	48	Y = 0.1250 × X + 4.000	0.9889	368.0000
	72	Y = 0.1150 × X + 22.00	0.7919	243.4783
	96	Y = 0.1650 × X + 13.00	0.8963	224.2424

Note: In the regression equation, X is the concentration, Y is the *Danio rerio* mortality, a is the slope of the equation, and b is the intercept of the equation.

**Table 2:** Effects of CuO-NPs with different particle sizes and morphologies on the body length of *Danio rerio* (mm).

Group	Treatment Time (d)			
	7	14	21	28
CK	26.78 ± 0.239 ^aA^	26.87 ± 0.403 ^aB^	26.73 ± 0.323 ^aAB^	26.92 ± 0.271 ^aA^
SP-L-L	26.78 ± 0.285 ^abA^	26.25 ± 0.287 ^abB^	26.52 ± 0.302 ^abAB^	26.95 ± 0.341 ^abA^
SP-L-H	26.84 ± 0.153 ^aA^	26.77 ± 0.225 ^aB^	26.72 ± 0.214 ^aAB^	26.34 ± 0.221 ^aA^
SP-S-L	26.99 ± 0.319 ^abA^	26.06 ± 0.331 ^abB^	26.50 ± 0.259 ^abAB^	26.73 ± 0.378 ^abA^
SP-S-H	26.77 ± 0.343 ^abA^	26.19 ± 0.292 ^abB^	26.38 ± 0.277 ^abAB^	26.49 ± 0.251 ^abA^
SC-L	26.89 ± 0.243 ^aA^	26.11 ± 0.244 ^aB^	26.84 ± 0.284 ^aAB^	26.73 ± 0.390 ^aA^
SC-H	26.61 ± 0.177 ^bA^	25.62 ± 0.435 ^bB^	25.86 ± 0.396 ^bAB^	26.09 ± 0.363 ^bA^

Note: Lowercase letters represent significant differences between treatment groups, and uppercase letters represent significant differences between treatment times.

**Table 3:** Effects of CuO-NPs with different particle sizes and morphologies on the body weight of *Danio rerio* (g).

Group	Treatment Time (d)			
	7	14	21	28
CK	0.347 ± 0.010 ^aA^	0.333 ± 0.008 ^aB^	0.330 ± 0.011 ^aB^	0.331 ± 0.010 ^aB^
SP-L-L	0.343 ± 0.016 ^abA^	0.329 ± 0.019 ^abB^	0.322 ± 0.016 ^abB^	0.316 ± 0.017 ^abB^
SP-L-H	0.338 ± 0.007 ^abA^	0.321 ± 0.007 ^abB^	0.321 ± 0.006 ^abB^	0.310 ± 0.007 ^abB^
SP-S-L	0.331 ± 0.012 ^bA^	0.302 ± 0.015 ^bB^	0.308 ± 0.020 ^bB^	0.298 ± 0.030 ^bB^
SP-S-H	0.335 ± 0.010 ^abA^	0.308 ± 0.009 ^abB^	0.309 ± 0.007 ^abB^	0.311 ± 0.006 ^abB^
SC-L	0.323 ± 0.011 ^abA^	0.321 ± 0.014 ^abB^	0.340 ± 0.017 ^abB^	0.305 ± 0.027 ^abB^
SC-H	0.344 ± 0.007 ^bA^	0.299 ± 0.011 ^bB^	0.283 ± 0.014 ^bB^	0.274 ± 0.009 ^bB^

Note: Lowercase letters represent significant differences between treatment groups, and uppercase letters represent significant differences between treatment times.

**Table 4:** Effects of CuO-NPs with different particle sizes and morphologies on the respiration rate of *Danio rerio* (times/30 s).

Group	Treatment Time (d)			
	7	14	21	28
CK	90 ± 1.09 ^aAB^	89 ± 2.61 ^aB^	92 ± 2.04 ^aA^	88 ± 1.77 ^aC^
SP-L-L	88 ± 2.97 ^abAB^	87 ± 4.59 ^abB^	97 ± 2.34 ^abA^	72 ± 3.69 ^abC^
SP-L-H	92 ± 1.03 ^aAB^	90 ± 1.27 ^aB^	100 ± 1.75 ^aA^	73 ± 3.47 ^aC^
SP-S-L	87 ± 2.12 ^abAB^	90 ± 1.78 ^abB^	91 ± 1.59 ^abA^	68 ± 5.92 ^abC^
SP-S-H	88 ± 2.01 ^bAB^	85 ± 1.51 ^bB^	92 ± 1.00 ^bA^	71 ± 5.01 ^bC^
SC-L	72 ± 4.96 ^dAB^	62 ± 3.56 ^dB^	60 ± 5.66 ^dA^	57 ± 3.16 ^dC^
SC-H	72 ± 3.89 ^Cab^	74 ± 4.07 ^cB^	72 ± 1.45 ^cA^	68 ± 4.98 ^cC^

Note: Lowercase letters represent significant differences between treatment groups, and uppercase letters represent significant differences between treatment times.

## Data Availability

The data that support the findings of this study are available on request from the corresponding author.
